# Needs for care of residents with schizophrenia spectrum disorders and association with daily activities and mood monitored with experience sampling method: the DIAPASON study

**DOI:** 10.1017/S2045796023000124

**Published:** 2023-04-11

**Authors:** Alessandra Martinelli, Miriam D'Addazio, Manuel Zamparini, Graham Thornicroft, Gabriele Torino, Cristina Zarbo, Matteo Rocchetti, Fabrizio Starace, Letizia Casiraghi, Mirella Ruggeri, Giovanni de Girolamo, the DiAPASon Collaborators

**Affiliations:** 1 Unit of Clinical Psychiatry, IRCCS Istituto Centro San Giovanni di Dio Fatebenefratelli, Brescia, Italy; 2 Unit of Epidemiological and Evaluation Psychiatry, IRCCS Istituto Centro San Giovanni di Dio Fatebenefratelli, Brescia, Italy; 3 Centre for Global Mental Health and Centre for Implementation Science, Institute of Psychiatry, Psychology and Neuroscience, King's College London, London, UK; 4 Department of Psychology, Clinical Psychology, Catholic University of the Sacred Heart, Milan, Italy; 5 Department of Mental Health and Dependence, ASST of Pavia, Pavia, Italy; 6 Department of Mental Health and Dependence, AUSL of Modena, Modena, Italy; 7 Section of Psychiatry, Verona Hospital Trust, AOUI, Verona, Italy

**Keywords:** Community mental health, epidemiology, psychiatric services, schizophrenia

## Abstract

**Aims:**

Care needs represent an essential paradigm in planning residential facility (RF) interventions. However, possible disagreements between users and staff are critical issues in service delivery. The Experience Sampling Method (ESM) tracks experiences in the real world and real time. This study aimed to evaluate the care needs of patients with schizophrenia spectrum disorder (SSD) in RFs and its association with daily activities and mood monitored using the ESM.

**Methods:**

As part of the DIAPASON project, 313 residents with SSD were recruited from 99 Italian RFs. Sociodemographic and clinical characteristics were recorded. Care needs, the severity of symptomatology and negative symptoms were assessed. Fifty-six residents were also assessed for 7 consecutive days using the mobile ESM. Descriptive, agreement, predictor and moderator analyses were conducted.

**Results:**

The staff rated a higher number of total and met needs than service users (*p* < 0.001). Only a slight agreement between users and staff on unmet needs was found in self-care (*k* = 0.106) and information (*k* = 0.100) needs, while a moderate agreement was found in accommodation (*k* = 0.484), food (*k* = 0.406), childcare (*k* = 0.530), physical health (*k* = 0.470), telephone (*k* = 0.458) and transport (*k* = 0.425) needs. Older age (−0.15; *p* < 0.01), longer SSD diagnosis (−0.16; *p* < 0.01), higher collaboration (−0.16; *p* < 0.01) and lower symptomatology (−0.16; *p* < 0.01) decreased the number of unmet needs, while being a female (0.27; *p* < 0.05) and a shorter length of stay in an RF (0.54; *p* < 0.001) increased the number of unmet needs. A higher number of unmet needs was associated with a lower amount of time spent in leisure activities or reporting a positive mood: on the contrary, more unmet needs were associated with a greater amount of time spent in religious or non-productive activities. The associations between unmet needs rated by staff and users and momentary mood as assessed using the ESM were not moderated by the severity of symptomatology.

**Conclusions:**

Although care needs are fundamental in planning residential activities aimed at recovery-oriented rehabilitation, RF interventions did not fully meet users' needs, and some disagreements on unmet needs between users and staff were reported. Further efforts are necessary to overcome Italian RF limits in delivering rehabilitative interventions defined by real users' needs to facilitate users' productivity and progress towards personal recovery.

## Introduction

The concept of mental health need has been suggested as a vital paradigm in planning mental health service interventions (Lasalvia *et al*., 2000) because it has direct treatment implications. Different definitions of mental health need have been suggested (Ruggeri *et al*., 2004), such as the public mental health need, assessed to provide services, programmes and staff to address this need, or the need for treatment of patients in specific mental health settings (e.g. a patient discharged from a psychiatric hospitalisation or receiving community psychiatric treatment). Furthermore, different assessment tools have been developed (Lasalvia *et al*., 2007; Campion *et al*., 2017; Mazzaia, 2018; Norman *et al*., 2018; Reisinger *et al*., 2021). In this study, we focus on the ‘need for care’, which indicates a perceived problem in a health or social domain of life (Ruggeri *et al*., 2004), as assessed by staff and patients with schizophrenia spectrum disorder (SSD) using the Camberwell Assessment of Need (CAN) to investigate a comprehensive range of health and social needs (Wennström and Wiesel, 2006).

A growing body of evidence has shown that mental health professionals and users may have different perceptions of needs for care (Slade *et al*., 1998; Brunt and Hansson, 2002; Ochoa *et al*., 2003). Patients and staff may disagree on both the presence of a need for care and on whether a need has or has not been met (Lasalvia *et al*., 2000; Cleary *et al*., 2006; Grinshpoon *et al*., 2008; Wiersma *et al*., 2009; Eklund and Ostman, 2010; de Girolamo *et al*., 2020). The disagreement between patients and professionals is a critical issue for service delivery, and any effort should be made to improve strategies aimed at increasing consensus between staff and patients, as a better staff–patient agreement may help improve treatment outcomes (Lasalvia *et al*., 2008).

Several studies have found an association between higher needs for care and specific sociodemographic variables, such as having a disability with severe symptomatology and low social functioning (Ruggeri *et al*., 2004). Moreover, needs for care are a better predictor of quality of life than clinical or sociodemographic variables, and are associated with patient-reported satisfaction with care (Ådnanes *et al*., 2019).

The Experience Sampling Method (ESM) tracks experiences in the real world and real-time (Granholm *et al*., 2008; Wee *et al*., 2019; Myin-Germeys and Kuppens, 2022) using self-reports to capture momentary experiences and their context. To date, several studies have used ESM in patients with SSD to evaluate daily mood or symptomatology (Myin-Germeys *et al*., 2001; Cho *et al*., 2017; Granholm *et al*., 2020); however, very few have used this methodology for assessing daily life activities of people with SSD (van Os *et al*., 2014; Kluge *et al*., 2018; Granholm *et al*., 2020; Culbreth *et al*., 2021). To the best of our knowledge, only van Os *et al*. (2014) have explicitly examined the association between needs for care evaluated via CAN and ecological indices monitored using the ESM in individuals with psychosis (Janney *et al*., 2013; van Os *et al*., 2014). They found an association between psychotic experiences and unmet needs moderated by negative affect (higher levels increased the number of unmet needs), positive affect (higher levels decreased the number of unmet needs) and environmental stress associated with events and activities (higher levels increased the number of unmet needs).

Community care in Italy is organised through 127 Departments of Mental Health that provide direct outpatient, hospital and residential care. In addition, many residential facilities (RFs) are managed by private (both non-profit and for-profit) organisations. All patients treated in private RFs are fully covered by the National Health Service for their stay and care. Previous studies have thoroughly assessed the residential care system (de Girolamo *et al*., 2002, 2005; Santone *et al*., 2005; Picardi *et al*., 2014).

RFs in Italy admit patients with SSD who have increased needs for care, severe psychopathology and low functioning (de Girolamo *et al*., 2002; Ministero Italiano della Salute, 2013; Martinelli *et al*., 2022*a*, 2022*b*), and who would have been hospitalised for a long time in a psychiatric hospital before de-institutionalisation (de Girolamo *et al*., 2002; Thornicroft and Tansella, 2004; McPhearson *et al*., 2018). RFs represent a fundamental component of long-term care and aim to support users in learning or relearning daily living skills and gaining confidence to achieve social inclusion, independent living and personal recovery (UN General Assembly, 2006; Priebe *et al*., 2009; Martinelli and Ruggeri, 2020*a*; Raugh *et al*., 2021), and to cover all adult roles (Ruggeri *et al*., 2004; Kimhy *et al*., 2014; McPhearson *et al*., 2018).

Despite the importance of RFs in Italian community mental health care after the early reform of psychiatric hospitals (Basaglia, 1968; Becker and Fangerau, 2018), they have not been surveyed for approximately 20 years (Culbreth *et al*., 2021). Furthermore, indeed the mission of Italian RFs to implement strategies aimed at developing rehabilitation activities oriented to the personal recovery ethos in order to increase personal daily life skills and well-being, and based on the real needs for care of residents (Junghan *et al*., 2007; Grinshpoon *et al*., 2008; Killaspy, 2016), no studies have investigated the association between needs for care and daily life activities in a sample of residents with SSD, in detail, using the ESM.

In the current study, we aimed to assess the needs for care as perceived by residents with SSDs and their key professionals, evaluate the agreement between users and staff perceptions of unmet needs, and identify sociodemographic and clinical predictors of unmet needs. Moreover, we aimed to address the gap in the literature by identifying unmet need predictors of daily activities and momentary mood as assessed using the ESM and investigating the interaction between symptomatology, momentary mood and unmet needs for care.

## Materials and methods

This study is part of the national project ‘DAily time use, Physical Activity, quality of care and interpersonal relationships in patients with Schizophrenia spectrum disorders (DIAPASON)’ (de Girolamo *et al*., 2020).

The DIAPASON project included 20 Departments of Mental Health and 17 private RFs in different Italian regions.

The inclusion criteria were: age between 20 and 55 years old, a DSM-5 diagnosis of SSD (American Psychiatric Association, 2013) and speaking and writing in the Italian language to participate adequately in a research interview. The exclusion criteria were: severe cognitive deficits (i.e. a Mini-Mental State Examination corrected score of <24), inability to provide informed consent, a recent (last 6 months) DSM-5 diagnosis of substance use disorder (American Psychiatric Association, 2013), a cerebrovascular/neurological disease and a history of clinically significant head injury.

In the participating RFs, the facility chiefs prepared an alphabetical list of patients with SSD on an index day, and based on this list, residents were consecutively invited to participate in the study until the recruitment target was achieved. From October 2020 to October 2021, 340 residents with SSD were recruited from 98 (public and private) RFs across Italy (12.8 ± 5.7 residents). Six patients (1.8%) were excluded due to severe cognitive deficits, and 21 (6.2%) dropped out after providing informed consent. A total of 313 patients were included in this study. Each RF recruited a mean of 3.5 (± 2.6) residents. Therefore, based on the total number of occupied beds, we recruited approximately 27% of the patients from each participating RF. The sample size calculation is described in detail in the study protocol (de Girolamo *et al*., 2020).

Due to logistic and financial limitations, the ecological ESM study was possible only in a sub-sample of RFs (*N* = 30); in these RFs, 56 (17.9%) residents were assessed using the ESM.

### Assessment of needs for care

Needs for care were assessed using the Italian version of the CAN (Phelan *et al*., 1995; Ruggeri *et al*., 1999), an interview developed for patients (CAN-P) and staff (CAN-S), comprising 22 items divided into five domains: health (physical health, psychotic symptoms, drugs, alcohol, safety to self, safety to others and psychological distress), basic (accommodation, food and daytime activities), social (sexual expression, social networks and intimate relationships), service (information, telephone, transport and benefits) and functioning (basic education, money, childcare, self-care and looking after the room). Each item was assessed on a three-point scale with 0 = no problem, 1 = no/moderate problem because of interventions (*met* need), and 2 = current serious problem (*unmet* need).

Users and their corresponding key professionals completed the two CAN versions in separate interviews.

### Assessment of the severity of symptomatology

The 24-item Brief Psychiatric Rating Scale (BPRS) (Overall and Gorham, 1962; Morosini and Casacchia, 1995) was used to assess symptom severity. The BPRS items were rated on a seven-point Likert scale ranging from 1 (no symptoms) to 7 (extremely severe symptoms) and divided into five categories (depression/anxiety, excitement, positive symptoms, negative symptoms and cognitive symptoms). The Brief Negative Symptom Scale (BNSS) (Strauss *et al*., 2012; Mucci *et al*., 2015) was used to assess the severity of negative symptoms. The BNSS items were rated on a six-point Likert scale ranging from 0 (no symptoms) to 6 (severe symptoms), and they evaluated blunted affect, alogia, asociality, anhedonia and avolition. For both the BPRS and BNSS, higher total mean scores indicated more severe symptomatology.

### Assessment of daily time use and emotions

Daily time use (i.e. daily activities) and emotions were assessed using a questionnaire on a smartphone-based application for ESM, developed ad hoc for the project (see the mobile application for ESM in online Supplementary materials). The mobile application comprised three sections: current activities, social contacts and mood. The first section asked, ‘*What are you doing right now*’? The participants could choose one or more of the following activity categories: sleeping; staying sick in bed; eating/self-care working; studying, doing housework; taking care of someone or something; voluntary working; doing leisure activities, thinking, resting, or doing nothing, performing sports or physical activity; getting around watching television or listening to the radio; and participating in religious activities (see list of daily activities in the online Supplementary Table 1).

The second section asked, ‘*Who are you with right now*’? and the participants could choose ‘alone’ or ‘with other people’. The third section showed seven mood conditions (happy, sad, tired, relaxed, nervous, quiet and full of energy) and asked the participants how they felt at that moment. The participants had to push on the screen and select the measure of that mood on a bar from 0 (not at all) to 100 (a lot).

Notifications appeared eight times a day, from 8:00 a.m. to 12:00 p.m., for 7 consecutive days. The notifications were semi-randomised (i.e. randomly sent within the scheduled time slots) in the following time slots: 8–10 a.m., 10 a.m.–12 p.m., 12 p.m.–2 p.m., 2–4 p.m., 4–6 p.m., 6–8 p.m., 8–10 p.m., 10 p.m.–12 a.m. A reminder notification appeared after 15 min. The participants had a maximum of 30 min to reply.

### Statistical analysis

Data were analysed using SAS, SPSS and R (R Core Team, 2013; SAS Institute Inc, 2013; IBM Corp. Released, 2020). Descriptive statistics comprised frequency tables for categorical variables and mean (standard deviation [SD]) for continuous variables. We tested the hypothesis of normality of continuous variables using the Kolmogorov–Smirnov test. To assess differences between matched user-rated and staff-rated needs for care, we used Wilcoxon's signed-rank test (because the variables were not normally distributed).

In addition to the number of needs (total, met and unmet), the met/unmet ratio was computed. Focusing only on unmet needs, we investigated the agreement between CAN-P and CAN-S on the total, domains and items using Cohen's *k* coefficient. According to Landis and Koch, the agreement is poor with *k* < 0.00, slight with *k* = 0.00–0.20, fair with *k* = 0.21–0.40, moderate with *k* = 0.41–0.60, substantial with *k* = 0.61–0.80 and almost perfect with *k* = 0.81–1.00 (McHugh, 2012).

To understand if the number of unmet needs (total and domains) could be associated with daily activities, we used generalised linear models (GLM) adjusted for age, sex and the BPRS total score.

With an additional statistical model, we tested the interaction between mood ratings collected through the ESM (momentary negative and positive mood considered as independent variables), the level of symptomatology severity (assessed through the BPRS, considered as a moderator) and unmet needs (both user-rated and staff-rated, considered as dependent variables).

To understand which clinical and/or sociodemographic factors could be associated with the difference between the unmet needs rated by the staff and users, we used GLM. Finally, as an external validation of our sample, we compared (through confidence intervals) the recorded percentages of unmet needs with those reported in similar previous studies (see online Supplementary Tables 2 and 3).

## Results

### Sociodemographic characteristics of the residents

As shown in Table 1, the users had a mean age of 41.0 years (s.d. = 9.7), and most users were males (70.3%), single (86.9%) and unemployed (83.3%). The mean length of mental disorder was 18.3 years (s.d. = 9.6), and they mostly had spent more than 5 years in the RF (43.9%).

**Table 1.** Socio-demographic and clinical characteristics of 313 residents of Italian RFs

	*N*	%
Sex		
Males	220	70.3
Females	93	29.7
Age (years)		
Mean (s.d.)	41.0	(9.7)
Marital status		
Single	271	86.9
Married or cohabiting	13	4.2
Divorced or widowed	28	9.0
Education level		
Elementary/junior high school	132	42.4
Secondary school/university degree	179	57.6
Working status^a^		
Working	38	12.2
Studying	14	4.5
Not working	260	83.3
Length of mental disorder (years)		
Mean (s.d.)	18.3	(9.6)
Length of stay in the rf (years)		
<1	53	39.1
1–5	122	17.0
>5	137	43.9
Support system		
Family/friends highly collaborative	72	23.1
Family/Friends interested but not supportive	134	42.9
Family/friends potentially available	50	16.0
Lack of social support	56	18.0
Collaboration skills		
Actively seek treatment, willing to collaborate	131	42.0
Wants to be helped, but lacks motivation	104	33.3
Passively accepts the treatment/intervention	45	14.4
Does not show attention or comprehension of treatment efforts	29	9.3
Actively refuses the treatment/intervention	3	1.0
BPRS score (range 1–7)	Mean	s.d.
Depression/anxiety	2.3	(0.9)
Excitement	1.8	(0.9)
Positive symptoms	2.4	(1.0)
Negative symptoms	2.3	(1.1)
Cognitive symptoms	1.8	(0.9)
Total score	2.1	(0.7)
BNSS score (range 0–6)		
Anhedonia	2.1	(1.6)
Distress	2.2	(1.8)
Asociality	2.2	(1.5)
Avolition	2.1	(1.6)
Blunted affect	1.9	(1.6)
Alogia	1.7	(1.6)
Total score	2.0	(1.3)

a ‘*Working*’ includes ‘full-time or part-time job in a protected environment’ and ‘protected environment job’. ‘*Studying*’ includes ‘job training course’, ‘student’. ‘*Not working*’ includes ‘housemaker’, ‘unemployed or looking for their first job’ and ‘retired who does not carry out any remuneration activity’ (including those who benefit from the invalidity pension).

Most patients' family/friends were available but not actively supportive (42.9%). Most users actively sought treatment (42%) or wanted to be helped but lacked motivation (33.3%). A few users (9.6%) performed no activities in the RFs, while the main activities performed were housekeeping (63.5%) and cleaning up (12.3%). The severity of symptomatology was mild (BPRS, mean total score 2.1 [range: 1–7; s.d. = 0.7] and BNSS, mean total score 2.0 [range: 0–6; s.d. = 1.3]) (Table 1).

### Differences in total, met and unmet needs between users and staff

The staff reported a significantly higher number of needs for care (*p* < 0.001) and met needs (*p* < 0.001) than users in all domains, except socially met needs (*p* = 0.138). Both the users and staff found the highest number of needs for care in health (users: 1.7; s.d. = 1.2 *v*. staff: 2.6; s.d. = 1.3) and the lowest in service (users: 0.9; s.d. = 0.9 *v*. staff: 1.0; s.d. = 1.0) (Table 2).

**Table 2.** Differences in total, met and unmet needs among 313 residents with SSD

	User-rated mean (s.d.)	Staff-rated mean (s.d.)	p*
Health			
Number of needs	1.7 (1.2)	2.6 (1.3)	**<0**.**001**
Met needs	1.2 (1.1)	1.9 (1.3)	**<0**.**001**
Unmet needs	0.5 (0.8)	0.7 (0.9)	**<0**.**001**
Ratio met/unmet	2.4	2.7	
Basic			
Number of needs	1.3 (1.0)	1.9 (1.0)	**<0**.**001**
Met needs	1.0 (0.9)	1.5 (1.0)	**<0**.**001**
Unmet needs	0.2 (0.5)	0.4 (0.7)	**<0**.**001**
Ratio met/unmet	5.0	2.5	
Social			
Number of needs	1.1 (1.0)	1.4 (1.0)	**<0**.**001**
Met needs	0.5 (0.6)	0.5 (0.7)	0.138
Unmet needs	0.6 (0.9)	0.9 (1.0)	**<0**.**001**
Ratio met/unmet	0.8	0.6	
Service			
Number of needs	0.9 (0.9)	1.0 (1.0)	**0**.**003**
Met needs	0.6 (0.8)	0.8 (0.8)	**<0**.**001**
Unmet needs	0.3 (0.6)	0.3 (0.5)	0.154
Ratio met/unmet	2.0	2.7	
Functioning			
Number of needs	1.2 (1.1)	2.1 (1.2)	**<0**.**001**
Met needs	1.0 (1.0)	1.6 (1.1)	**<0**.**001**
Unmet needs	0.2 (0.6)	0.5 (0.9)	**<0**.**001**
Ratio met/unmet	5.0	3.2	
Total			
Number of needs	6.1 (3.6)	9.0 (3.8)	**<0**.**001**
Met needs	4.2 (2.8)	6.3 (3.1)	**<0**.**001**
Unmet needs	1.9 (1.8)	2.7 (2.7)	**<0**.**001**
Ratio met/unmet	2.2	2.3	

Scoring of CAN items: 0 = no problem, 1 = no/moderate problem because of continuing interventions (met need) and 2 = current serious problem whether or not help is offered or given (unmet needs ratio ⩾ 1 indicates a proportion between met and unmet needs in favour of met needs). Bold values denote statistical significance at the *p* < 0.05 level.

The overall ratio was similar between the two groups (total: users 2.2 *v*. staff 2.3). The highest differences in the ratio among the users and staff were found in basic (ratio: users 5.0 *v*. staff 2.5) and functioning (ratio: users 5.0 *v*. staff 3.2) needs (Table 2).

### Percentage of agreement on unmet needs between users and staff

Of the 313 user–staff pairs included in the analyses, 175 (55.9%) of the user–staff pairs reported unmet needs (Table 3). The highest number of unmet needs was reported by the staff and users in social (98; 31.3) needs, while the lowest was in functioning needs for users (55; 17.6) and service needs for the staff (68; 21.7) (Table 3).

**Table 3.** Number of unmet needs (rating 2) identified by patients, staff and patient ± staff pairs and total percentage agreement for each can item

CAN categories	User-rated unmet needs *N* (%)	Staff-rated unmet needs *N* (%)	User/staff-rated pairs of unmet needs *N* (%)	Total percentage agreement (*k* di Cohen)
Total	202 (64.5)	239 (76.4)	175 (55.9)	0.313
*Basics*	62 (19.8)	100 (32.0)	40 (12.8)	0.330
Accommodation	28 (9.0)	32 (10.2)	16 (5.1)	0.484
Food	22 (7.0)	39 (12.5)	14 (4.5)	0.406
Daytime activities	26 (8.3)	59 (18.9)	16 (5.1)	0.295
*Social*	125 (39.9)	171 (54.6)	98 (31.3)	0.373
Company	69 (22.0)	122 (39.0)	48 (15.3)	0.308
Intimate relationships	69 (22.0)	99 (31.6)	45 (14.4)	0.373
Sex life	55 (17.6)	53 (16.9)	25 (8.0)	0.351
*Functioning*	55 (17.6)	101 (32.3)	41 (13.1)	0.386
Basic education	1 (0.3)	6 (1.9)	1 (0.3)	0.282
Money	35 (11.2)	59 (18.9)	21 (6.7)	0.357
Childcare	8 (2.6)	14 (4.5)	6 (1.9)	0.530
Self-care	9 (2.9)	32 (10.2)	3 (1.0)	0.106
Living environment	20 (6.4)	46 (14.7)	10 (3.2)	0.235
*Health*	101 (32.3)	141 (45.1)	76 (24.3)	0.404
Physical health	18 (5.8)	18 (5.8)	9 (2.9)	0.470
Psychotic symptoms	44 (14.1)	95 (30.4)	32 (10.2)	0.332
Drugs	0	4 (1.3)	0	
Alcohol	1 (0.3)	3 (1.0)	0	
Safety to self	12 (3.8)	5 (1.6)	2 (0.6)	0.218
Safety to others	2 (0.6)	5 (1.6)	1 (0.3)	0.279
Psychological distress	71 (22.7)	85 (27.2)	42 (13.4)	0.387
*Services*	77 (24.6)	68 (21.7)	38 (12.1)	0.381
Information	27 (8.6)	13 (4.2)	3 (1.0)	0.100
Telephone	13 (4.2)	20 (6.4)	8 (2.6)	0.458
Transport	30 (9.6)	36 (11.5)	16 (5.1)	0.425
Benefits	27 (8.6)	13 (4.2)	8 (2.6)	0.364

We did not find any substantial or almost perfect agreement on unmet needs between staff and users. The highest agreement was moderate for accommodation (*k* = 0.484), food (*k* = 0.406), childcare (*k* = 0.530), physical health (*k* = 0.470), telephone (*k* = 0.458) and transportation (*k* = 0.425). A slight agreement was found in self-care (*k* = 0.106), where mental health professionals rated lower than patients, and information (*k* = 0.100), where mental health professionals rated higher than patients. A fair agreement was found for the other CAN items (Table 3).

### Predictors of differences of unmet needs between users and staff

As shown in online Supplementary Table 3, negative associations were found between the following variables: social unmet needs and age (older age decreased the number of unmet needs) (*β* = −0.15; *p* < 0.01), length of mental health disorder (longer diagnosis of SSD decreased the number of unmet needs) (*β* = −0.16; *p* < 0.01), functioning unmet needs and collaboration skills (higher collaboration decreased the number of unmet needs) (*β* = −0.16; *p* < 0.01), BPRS (lower symptomatology decreased the number of unmet needs) (*β* = −0.17; *p* < 0.01), total unmet needs and collaboration skills (higher collaboration decreased the number of unmet needs) (*β* = −0.12; *p* < 0.05) and BPRS (shorter symptomatology decreased the number of unmet needs) (*β* = −0.16; *p* < 0.01).

Positive associations were found between unmet health needs and sex (being a female increased the number of unmet needs) (*β* = 0.27; *p* < 0.05), social unmet needs and length of stay in the RF (shorter length of stay in the RF increased the number of unmet needs), particularly when the length of stay was <1 year (*β* = 0.54; *p* < 0.001) and between 1 and 5 years (*β* = 0.36; *p* < 0.1) (online Supplementary Table 4).

### User-rated and staff-rated unmet need predictors of activities and momentary mood as measured using the ESM

As shown in Table 4, among the predictors, negative associations were similarly found in users and staff between leisure activities and health (users: *β* = −0.22; *p* < 0.05 *v*. staff: *β* = −0.33; *p* < 0.05) and total unmet needs (users: *β* = −0.29; *p* < 0.05 *v*. staff: *β* = −0.11; *p* < 0.05) (higher number of unmet needs decreased leisure activities); positive mood and unmet health needs (users: *β* = −0.41; *p* < 0.001 *v*. staff: *β* = −0.32; *p* < 0.05). Only for users, there was a negative association between leisure activities and social (*β* = −0.29; *p* < 0.05) and service unmet needs (*β* = −0.28; *p* < 0.05) (higher number of unmet needs decreased leisure activities), and positive mood and total unmet needs (*β* = −0.62; *p* < 0.001) (higher number of unmet needs was associated with lower positive mood). Only for the staff, there was a negative association between leisure activities and basic needs (*β* = −0.41; *p* < 0.05) (a higher number of unmet needs decreased leisure activities).

**Table 4.** User-rated (CAN-P) and staff-rated (CAN-S) domains unmet needs as predictors of activities and momentary mood (negative and positive affect) as measured with ESM

	Dependent variables							
Predictors*	Non-productive activities	Productive activities	Leisure activities	Physical activities	Self-care	Religious activities	Positive affect	Negative affect
User-rated (CAN-P)								
Basic	0.07 (−0.16; 0.30)	−0.17 (−0.39; 0.06)	0 (−0.22; 0.23)	−0.03 (−0.26; 0.20)	0.01 (−0.22; 0.24)	0.03 (−0.20; 0.26)	−0.02 (−0.26; 0.21)	−0.05 (−0.28; 0.18)
Social	−0.01 (−0.28; 0.27)	−0.25 (−0.52; 0.01)	−0.29* (**−0.56; −0.03)**	−0.25 (−0.51; 0.02)	−0.11 (−0.38; 0.17)	−0.22 (−0.49; 0.05)	−0.26 (−0.53; 0.01)	0.08 (−0.20; 0.35)
Functioning	0.27** (**0.08; 0.47)**	−0.09 (−0.31; 0.12)	−0.06 (−0.27; 0.15)	−0.15 (−0.35; 0.06)	0.01 (−0.20; 0.22)	−0.13 (−0.34; 0.08)	−0.16 (−0.37; 0.05)	0.20 (−0.01; 0.41)
Health	0.14 (−0.06; 0.34)	−0.13 (−0.33; 0.07)	−0.22* (**−0.41; −0.03)**	−0.13 (−0.33; 0.07)	0.05 (−0.16; 0.25)	−0.09 (−0.29; 0.11)	−0.41*** (**−0.59; −0.24)**	0.38*** (**0.21; 0.56)**
Services	0.17 (−0.10; 0.44)	0.10 (−0.17; 0.37)	−0.28* (**−0.54; −0.02)**	0.07 (−0.20; 0.34)	0.02 (−0.26; 0.30)	0.09 (−0.18; 0.37)	−0.15 (−0.91; 0.25)	0.04 (−0.23; 0.31)
Total	0.23 (−0.01; 0.46)	−0.21 (−0.44; 0.03)	−0.29* (**−0.52; −0.07)**	−0.19 (−0.43; 0.04)	0 (−0.24; 0.25)	−0.14 (−0.38; 0.10)	−0.40*** (**−0.62; −0.18)**	0.29* (**0.07; 0.52)**
Staff-rated (CAN-S)								
Basic	0.07 (−0.16; 0.30)	−0.03 (−0.46; 0.40)	−0.41* (**−0.82; −0.01)**	0.26 (−0.16; 0.67)	0.10 (−0.33; 0.53)	0.45* (**0.04; 0.86)**	−0.21 (−0.64; 0.22)	0.19 (−0.23; 0.62)
Social	0.13 (−0.16; 0.41)	−0.14 (−0.43; 0.15)	−0.04 (−0.33; 0.24)	−0.09 (−0.37; 0.20)	0.07 (−0.22; 0.36)	−0.08 (−0.37; 0.21)	−0.12 (−0.41; 0.17)	0.22 (−0.06; 0.50)
Functioning	0.39* (**0.04; 0.73)**	0.13 (−0.23; 0.49)	−0.16 (−0.51; 0.19)	0.20 (−0.16; 0.55)	0.02 (−0.34; 0.38)	0.18 (−0.18; 0.53)	−0.25 (−0.60; 0.11)	0.21 (−0.14; 0.57)
Health	0.20 (−0.10; 0.50)	−0.17 (−0.47; 0.14)	−0.33* (**−0.62; −0.04)**	−0.11 (−0.42; 0.19)	0.27 (−0.03; 0.57)	−0.06 (−0.37; 0.25)	−0.32* (**−0.62; −0.02)**	0.39** (**0.10; 0.68)**
Services	−0.32 (−0.97; 0.32)	−0.18 (−0.83; 0.47)	−0.43 (−1.06; 0.21)	0.25 (−0.39; 0.90)	0.26 (−0.39; 0.91)	0.34 (−0.30; 0.99)	0.25 (−0.41; 0.90)	0.02 (−0.62; 0.67)
Total	0.08 (−0.03; 0.19)	−0.04 (−0.15; 0.07)	−0.11* (**−0.22; −0.01)**	0.02 (−0.10; 0.13)	0.06 (−0.05; 0.18)	0.04 (−0.07; 0.15)	−0.09 (−0.20; 0.02)	0.12* (**0.01; 0.23)**

A linear regression, adjusted for age, sex and BPRS (standardised coefficients), was undergone. Bold values denote statistical significance at *p < 0.05 **p < 0.01 ***p < 0.001 level.

A positive association in both user and staff ratings was found between non-productive activities and functioning (users: *β* = 0.27; *p* < 0.01 *v*. staff: *β* = 0.39; *p* < 0.05) (higher number of unmet needs were associated to higher non-productive activities), negative mood and health (users: *β* = 0.38; *p* < 0.001 *v*. staff *β* = 0.39; *p* < 0.01) and total unmet needs (users: *β* = 0.29; *p* < 0.05 *v*. staff: *β* = 0.12; *p* < 0.05) (higher number of unmet needs was associated with more negative mood). Only in staff ratings was there a positive association between religious activities and basic needs (*β* = 0.45; *p* < 0.05) (a higher number of unmet needs increased religious activities) (Table 4).

### Association between momentary mood (as assessed using the ESM) and user-rated and staff-rated unmet needs at different levels of the BPRS

**Figure 1.**
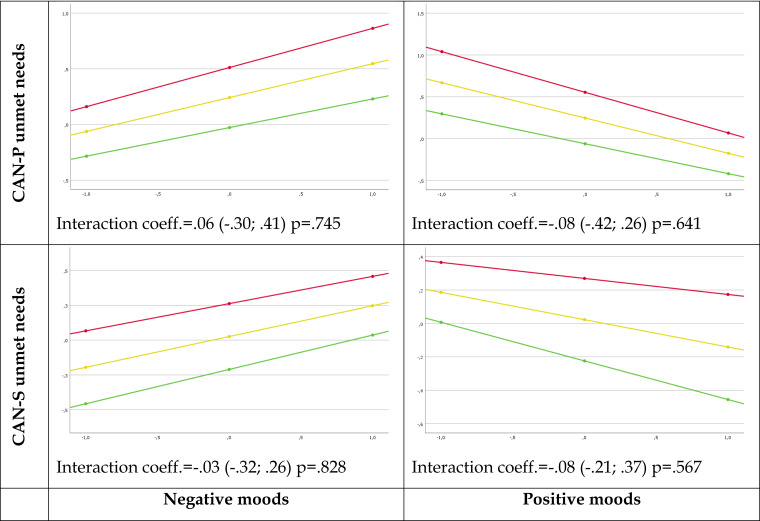
Plot of the simple slope analysis for the moderator variable BPRS: association between mood ratings (positive affect and negative affect as assessed with ESM) and user-rated (CAN-P) and staff-rated (CAN-S) unmet needs at different severity levels of BPRS (the lowest symptomatology severity: green line, intermediate symptomatology severity: yellow line, the highest symptomatology severity: red line).

As shown in Fig. 1, a higher number of user-rated and staff-rated unmet needs negatively influenced users' positive moods and positively influenced users' negative moods. However, the associations between user-rated and staff-rated unmet needs and momentary mood (negative and positive), as assessed using the ESM, were not moderated by the level of severity of symptomatology, as assessed using the BPRS (negative mood and number of unmet needs in CAN-P [interaction coefficient = 0.06 (−0.30; 0.41), *p* = 0.745], and CAN-S [interaction coefficient = −0.03 (−0.32; 0.26), *p* = 0.828]; positive mood and the number of unmet needs in both CAN-P [interaction coefficient = −0.08 (−0.42; 0.26), *p* = 0.641] and CAN-S [interaction coefficient = −0.08 (−0.21; 0.37), *p* = 0.567]).

## Discussion

This was the first study to investigate the association between needs for care and ecological indices, as assessed using the ESM, in residents with SSD. We confirmed the sociodemographic and clinical characteristics of similar samples from previous surveys (de Girolamo *et al*., 2002; Martinelli *et al*., 2022*b*). Our findings reveal that users who were younger, females, with the most severe symptomatology, the shortest length of stay in an RF or without collaborative behaviour were the most likely to report unmet needs in social, functioning and health areas. This feature might be related not only to the natural course of SSD with reduced symptoms and increased psychosocial functioning when patients are more collaborative, but also to the process of ‘institutionalisation’ (Wennström and Wiesel, 2006; SAS Institute Inc, 2013; Martinelli *et al*., 2019) which implies limitations in their social reintegration in society. The longer their stay in an RF, the higher the risk of being socially isolated (Federici *et al*., 2009; Gold, 2014; Martinelli *et al*., 2022*a*).

Our findings reveal that the agreement between users and professionals concerning needs for care (Phelan *et al*., 1995) is currently a challenge for Italian RFs. Moderate agreement was reported in areas where it was easier to allocate resources, such as housing and daytime activities (Werner, 2012), which represent the most frequent activities carried out by the residents of our sample. The slight agreement on self-care, which usually represents one of the main objectives in RFs, might be due to an overestimation of self-care by users with SSD, who frequently have negative symptoms which may impair their body perception and lead to neglecting personal hygiene (Goldstone, 2020). Furthermore, although the staff reported that patients were adequately informed about their disorder and ongoing treatments, users evaluated this information as not comprehensive. This might be due to staff overestimation of their communication skills or to the effect of self-stigma in patients' reticence about asking for more information (Lanfredi *et al*., 2015; Winkler *et al*., 2017; Atwoli and Muhia, 2021). The finding of a relatively close agreement on unmet needs concerning social life (sex life in particular) was somewhat surprising, considering that this area may otherwise be challenging to investigate (Ruggeri *et al*., 2004). However, the highest unmet needs in social areas domain confirmed (Eklund and Ostman, 2010; Gold, 2014) that few services are able to fulfil personal and subjective needs. A moderate disagreement was reported between staff and users on functioning, probably because most residents collaborated in the rehabilitative programme, which positively balanced the number of unmet needs in this area.

Most users performed some kinds of activity in the RF. However, the increase in unmet needs influenced the level of performed activity. In particular, the lack of productive activities strongly correlated with high unmet needs in functioning, while leisure activities seemed to be the first activities not performed when there was an overall increase in unmet needs, particularly in physical and psychological health, social life, media and transport.

Higher levels of met needs in physical and psychological health were associated with an increase in positive mood. Furthermore, a higher number of unmet needs were negatively associated with the fulfilment of everyday productive activities. These features may hinder the achievement of users' recovery because the lesser the user is active, motivated to pursue a productive activity, and proactive, the lesser they will have to cover all adult roles, live independently and be socially integrated (Argentzell *et al*., 2020).

Interestingly, our findings reveal that the association between mood and unmet needs is not moderated by the severity of symptomatology, leading us to conclude that needs for care, and particularly unmet needs, represent an important feature to be considered when planning residential interventions, independent of the severity of symptoms (Grinshpoon and Ponizovsky, 2008; Oorschot *et al*., 2012; Salisbury *et al*., 2016).

### Strengths and limitations

A major strength of this study is the use of the ESM, which allows the collection of longitudinal, prospective data in real-time, reducing reporting biases and acquiring information that cannot be easily observed or monitored in daily life.

The possible assessment bias due to staff socio-demographics was reduced thanks to a comprehensive staff training on the use of the CAN and other assessment tools.

Although residents with SSD represent most of those living in RFs (Starace *et al*., 2017), these findings cannot be generalised to residents with other diagnostic profiles or those with SSD also showing marked cognitive impairment.

A limitation of the study was that because of logistic and financial limitations, the use of ESM was possible only in 17.9% of enrolled residents living in a subgroup of RFs, therefore reducing the generalisation of our findings.

Another limitation was the lack of a detailed statistical analysis plan for this specific CAN investigation in the study protocol (de Girolamo *et al*., 2020).

We were also not able to perform a detailed statistical analysis, such as to cluster the sample, particularly the ESM subsample, based on the RFs where they live because of the limitation in the overall sample size.

Finally, data were collected during the coronavirus disease 2020 pandemic, which influenced daily clinical practice and routine activities.

## Conclusions

This is the first study to investigate the needs for care of residents with SSD and its association with daily activities and mood, as monitored using the ESM.

Our findings reveal that although needs for care are important for planning rehabilitative activities (Lasalvia *et al*., 2008; National Institute for Health and Care Excellence, 2020; Martinelli and Ruggeri, 2020*a*), Italian RFs deliver interventions which do not fully meet them. Furthermore, despite national and international guidelines (Grinshpoon and Ponizovsky, 2008; IBM Corp. Released, 2020; Martinelli and Ruggeri, 2020*b*) recommending the implementation of shared decision-making to promote users' recovery, we found a substantial disagreement concerning unmet care needs between users and staff. Hence, Italian RFs need to deliver rehabilitative interventions that match real users' needs for care to facilitate their productive activities and progress towards recovery.

Further studies are needed to evaluate whether the use of the ESM might facilitate the design of tailored rehabilitative interventions based on the consensus of users and staff regarding needs for care.

## Data Availability

Dataset referring to this manuscript is published with restricted access on Zenodo platform and accessible at this link: https://doi.org/10.5281/zenodo.686604.

## Supplementary material

For supplementary material accompanying this paper visit http://doi.org/10.1017/S2045796023000124. click here to view supplementary material
