# Needs for care of residents with schizophrenia spectrum disorders and association with daily activities and mood monitored with experience sampling method: the DIAPASON study – CORRIGENDUM

**DOI:** 10.1017/S2045796025100097

**Published:** 2025-07-01

**Authors:** Alessandra Martinelli, Miriam D'Addazio, Manuel Zamparini, Graham Thornicroft, Gabriele Torino, Cristina Zarbo, Matteo Rocchetti, Fabrizio Starace, Letizia Casiraghi, Mirella Ruggeri, Giovanni de Girolamo, the DiAPASon Collaborators

An unauthorised version of the Italian MMSE was used by the study team without permission; however, this has now been rectified with PAR. The MMSE is a copyrighted instrument and may not be used or reproduced in whole or in part, in any form or language or by any means without written permission of PAR (www.parinc.com).

The authors apologise for the error.
