# Supplementary material for: Needs for care of residents with schizophrenia spectrum disorders and association with daily activities and mood monitored with experience sampling method: the DIAPASON study
Source: Epidemiol Psychiatr Sci. 2023 Apr 11;32:e18. doi: 10.1017/S2045796023000124 (PMC10130736; doi:10.1017/S2045796023000124)
Supplement: Supplementary file 1 [file S2045796023000124sup001.docx]

**SUPPLEMENTARY MATERIALS**

**DESCRIPTION OF THE MOBILE APPLICATION FOR ESM**

**D**aily time use (i.e., daily activities) and emotions were assessed using a questionnaire on a phone-based application for ESM. The ESM questionnaire, developed ad hoc for the project, was based on the ISTAT questionnaire and evaluated some of the variables investigated in the adapted ISTAT questionnaire as current activity, perceived energy level, and mood state (Merikangas et al., 2019).

In order to create the ESM questionnaire, initially it was important to identify the items we wanted to include and once the different items were identified, we assembled them into a short coherent questionnaire. Regarding the formulation of the items, we decided to follow Germeys & Kuppens guidelines.

About the timeframe, we decided to refer the questions to the present moment (“What are you doing right now?” and “Who are you with right now?" – Section 1; **"**Right now, I feel…." – Section 2). This time reference reduces recall bias to a minimum, it is an advantage of capturing every single aspect of every moment in daily life and improves the clarity of the questions making the momentary nature of the assessment explicit (i.e. “How happy are you?” could refer to the momentary happiness but also overall satisfaction with life) (Germeys & Kuppens, 2021). Moreover, every question of ESM items complied with *the checklist for optimally formulated ESM question suggested* to Germeys & Kuppens. Specifically, every question about daily time use and mood:

- were short and to the point, to ensure that each question was easy to read, fits the screen of the mobile device well and can be quickly answered;
- avoided extreme wording in order to be as clear as possible;
- avoided negative wording to not put participants in a negative mindset;
- avoided jargon in order to help participants to use themselves to describe a concept;
- used answers that reflect global self-views;
- was relevant in any kind of context and could be answered in any type of situation.

The responses to the selected questions were based on selected options for the measure of daily time used and on a continuous scale to evaluate mood. The continuous scale has the advantage to allow higher precision than discrete scales because participants can assign a score between two answer options. Most studies have found largely equivalent reliability and validity for continuous rating scales (Gries et al., 2017; Kuhlmann et al., 2017; Lukacz et al., 2004) and the literature suggests questionnaire lengths range from 1 to over 100 (Morren et al., 2009; Ono et al., 2019; Vachon et al., 2019), that we adopted.

At a later time, the ESM was downloaded on the smartphone of all the participants and each participant received a short 30-minute training on the use of ESM procedures. They received a smartphone and researchers asked to bring it with them for a period of 7 days: the smartphone generated a "recall" acoustic signal and sent an SMS 6-8 times a day, at random intervals, between 9:00 and 21:00. Participants had to complete the ESM questionnaire on the smartphone screen, it took about 3-4 minutes and the questionnaires must be completed no later than 30 minutes after the request, as suggested to the literature guidelines in order to keep the questionnaire as short as possible for minimize the interruptions to the participant’s daily lives (Kimhy et al., 2020; Eisele et al., 2020).

We opted for semi-randomized notifications (i.e., randomly sent within the scheduled time slots) and signal contingent, which means that push notifications appear to signal participants to fill out the questionnaire. The notifications were semi-randomized because, according to Germays & Kuppens (2021), keeping the order constant can make the assessments feel repetitive and boring, it could also allow participants to answer faster, which may reduce the burden, indeed randomizing the order of questions can prevent these effects from introducing bias to the data. As reported by De Girolamo et al.., about the Diapason project, the usability and adherence of ESM in clinical populations and healthy subjects were good (De Girolamo et al., 2022).

References

Eisele, G., Vachon, H., Myin-Germeys, I., & Viechtbauer, W. (2021). Reported affect changes as a function of response delay: Findings from a pooled dataset of nine experience sampling studies. *Frontiers in Psychology*. doi:10.3389/fpsyg.2021.580684

Gries, K., Berry, P., Harrington, M., Crescioni, M., Patel, M., Rudell, K., Vernon, M. (2017).

Literature review to assemble the evidence for response scales used in patient-reported outcome measures. *J Patient Rep Outcomes, 2*, 41. doi:10.1186/s41687-018-0056-3

Kimhy, D., Myin-Germeys, I., Palmier-Claus, J., & Swendsen, J. (2012). Mobile assessment guide for research in schizophrenia and severe mental disorders. *Schizophr Bull, 38*(3), 386-395. doi:10.1093/schbul/sbr186

Kuhlmann, T., Dantlgraber, M., & Reips, U. D. (2017). Investigating measurement equivalence of visual analogue scales and Likert-type scales in Internet-based personality questionnaires. *Behav Res Methods,49*(6), 2173-2181. doi:10.3758/s13428-016-0850-x

Lukacz, E. S., Lawrence, J. M., Burchette, R. J., Luber, K. M., Nager, C. W., & Buckwalter, J. G. (2004). The use of Visual Analog Scale in urogynecologic research: a psychometric evaluation. *Am J Obstet Gynecol,191*(1), 165-170. doi:10.1016/j.ajog.2004.04.047

Merikangas KR, Swendsen J, Hickie IB, Cui L, Shou H, Merikangas AK, et al. Real-time mobile monitoring of the dynamic associations among motor activity, energy, mood, and sleep in adults with bipolar disorder. JAMA Psychiatry. 2019;76(2):190–198

Myin - Germeys & Kuppens. The Open Handbook of Experience Sampling Methodology: A step-by-step guide to designing, conducting, and analyzing ESM studies Center for Research on Experience Sampling and Ambulatory Methods Leuven (REAL), 2021

Morren, M., van Dulmen, S., Ouwerkerk, J., & Bensing, J. (2009). Compliance with momentary pain measurement using electronic diaries: a systematic review. *Eur J Pain, 13*(4), 354-365. doi:10.1016/j.ejpain.2008.05.010

Ono, M., Schneider, S., Junghaenel, D. U., & Stone, A. A. (2019). What Affects the Completion of Ecological Momentary Assessments in Chronic Pain Research? An Individual Patient Data Meta-Analysis. *J Med Internet Res,21*(2), e11398. doi:10.2196/11398

Vachon, H., Viechtbauer, W., Rintala, A., & Myin-Germeys, I. (2019). Compliance and Retention With the Experience Sampling Method Over the Continuum of Severe Mental Disorders: Meta-Analysis and Recommendations. *J Med Internet Res, 21*(12), e14475. doi:10.2196/14475

Zarbo C, Agosta S, Casiraghi L, De Novellis A, Leuci E, Paulillo G, Rocchetti M, Starace F, Zamparini M, de Girolamo G. Assessing adherence to and usability of Experience Sampling Method (ESM) and actigraph in patients with Schizophrenia Spectrum Disorder: A mixed-method study. Psychiatry Res. 2022 Aug;314:114675. doi: 10.1016/j.psychres.2022.114675. Epub 2022 Jun 11. PMID: 35751998.

**1.1 MOBILE APPLICATION FOR MONITORING**

**USE OF TIME AND MOOD**

***DiAPAson Project***

**APP display:**

A page for each question

**Notifications:**

- 8 times a day, from 8.00 am to 12.00 pm in the following time slots: 8-10 am, 10-1 am, 12 am-2 pm, 2-4 pm, 4-6 pm, 6-8 pm, 8-10 pm, 10-12 pm.
- Notifications are randomly sent within the scheduled time slots.
- A reminder is expected after 15 minutes.
- The participant has to respond within 30 minutes.

**SECTION 1**

**1) What are you doing right now?**

*(You can also select more options)*

- Sleeping
- Stay sick in bed
- Eat / drink / breakfast / snack
- Self-care (washing, dressing, etc.)
- Work or do internship / internship (or look for work)
- Studying / attending training courses
- Cleaning, cooking, tidying up the house or car, shopping
- Take care of someone or something (people, animals, plants)
- Voluntary work
- Leisure activities (e.g. social life, playing, chatting, reading, going to the cinema, playing an instrument, etc.)
- Thinking, resting, doing nothing (without sleeping)
- Doing sports, physical activity
- Getting around (on foot or by bicycle, car, public transport)
- Watch TV / listen to the radio
- Participate in religious activities (e.g. go to mass, pray, etc.)

**2) Who are you with right now?**

*(Only one option can be selected)*

- Alone
- With other people

**SECTION 2**

*(These 6 questions are shown in a random order)*

**Right now, I feel….**

**1) Happy**

--> bar 0-100 (0 not at all - 100 a lot)

**2) Sad**

--> bar 0-100 (0 not at all - 100 a lot)

**3) Tired**

--> bar 0-100 (0 not at all - 100 a lot)

**4) Relaxed**

--> bar 0-100 (0 not at all - 100 a lot)

**5) Nervous**

--> bar 0-100 (0 not at all - 100 a lot)

**6) Quiet**

--> bar 0-100 (0 not at all - 100 a lot)

**7) Full of energy**

--> bar 0-100 (0 not at all - 100 a lot)

**SECTION 3**

*(Question added only in the time slot 6-8 pm: it follows the other questions)*

**1) Thinking about today, would you say it was a typical day for you?**

- Yes
- No

If the participant clicks NO, they will see the following message:

**Specify the reason: ___________________________________________________________________** (possibility to enter free text

## SUPPLEMENTARY TABLE 1S

## GUIDELINE LIST OF DAILY ACTIVITIES ASSESSMENT

| **Activity** | **Activity description** |
| --- | --- |
| **Sleeping** | *Moments when you were sleeping, light or deep sleep.* |
| **Staying sick in bed** | *Moments when you were awake sick in the bed.* |
| **Eating** | *Meal-related activities, such as eating, drinking, having lunch, dinner, snacking, drinking.* |
| **Self-caring** | *Self-care activities, such as washing, dressing, combing, taking medical care (e.g., having a medical examination, taking medicine).* |
| **Working** | *Work-related activities (and lunch breaks or breaks during work) or looking for work.* |
| **Studying** | *Activities related to school or training, such as taking courses or lessons, taking exams, studying, doing homework.* |
| **Doing houseworks** | *Activities related to the management of your home or personal property (such as a car), such as: cooking, washing (e.g. dishes, house, clothes), tidying up, cleaning, sewing, ironing, building and renovating the house (e.g. painting the walls, repairing something in the house), shopping, buying services (e.g. electricity, gas), going to the hairdresser / barber, managing family life.* |
| **Taking care of someone or something** | *Activities related to the care of one's family (adults or children), animals or plants: for example, helping or playing with children, providing physical care, or keeping company); do gardening (care of plants, vegetable garden, flowers); taking care of animals (feeding, walking).* |
| **Voluntary working** | *Voluntary work carried out within a group / association, or aid given free of charge to people from other families (including children not living together).* |
| **Doing leisure activities** | *Leisure activities, such as going to the cinema, theatre, concert, exhibition or museum, taking cultural trips, painting, photographing, making videos, playing an instrument, writing poetry, making collections, using e-mail (not for work), search for information on the internet, play (even on the PC or with video games, or with animals), read (newspapers, books, magazines), socialize, make and receive visits, text messages, converse on the phone, celebrate, chat with someone.* |
| **Resting, doing nothing** | *Activities of relaxation, rest, thinking or meditating, doing nothing (without sleeping), smoking.* |
| **Doing physical activity** | *Leisure activities that require physical exercise such as sports, dancing, walking, strolling, running, playing with the ball, fitness, hunting, fishing, mushroom / plant picking, and all outdoor sports activities.* |
| **Getting around** | *Moments when you were moving.* |
| **Watching TV or listening to the radio** | *Leisure activities such as watching television, videos, TV series, movies, or listening to the radio / music.* |
| **Participating to religious activities** | *Activities of participation in religious activities, such as religious meetings / gatherings or religious ceremonies, praying, going to a place of worship, going to the cemetery.* |

**SUPPLEMENTARY TABLE 2S**

**BASIC CHARACTERISTICS OF OTHER STUDIES DATA USING BOTH USERS’ AND STAFF’ RATINGS ON CAN IN PATIENTS WITH SSD**

| **Author & year** | **Title** | **Patients** | **Mean age** | **Gender** | **Duration of mental disorder** |
| --- | --- | --- | --- | --- | --- |
| Cleary & Hunt., 2006 | The patient’s view of need and caregiving consequences: a cross-sectional study of inpatients with severe mental illness | N= 85 with schizophrenia, hospitalized or residential | 37 | M 55, F 30 | 7 (<1 year), 36 (1-5 years),  42 (>5 years) |
| Brunt & Hansson, 2002 | Comparison of user assessed needs for care between psychiatric inpatients and supported community residents | N=75: 51 residents (even light support) and 36 diagnosis of psychosis | 41 | M 29, F 22 | 19 yrs |
| Grinshpoon & Ponizovsky, 2008 | The relationships between need profiles, clinical symptoms, functioning and the well-being of inpatients with severe mental disorders | N=52, with SSD | 41.5 | M 34, F 18 | 14 yrs |
| Martinelli et al., 2022 | Performance and effectiveness of step progressive care pathways within mental health supported accomodation services in Italy | N=161 with psychosis; N=116 with mood disorders, or personality disorders | 48.2 | M 102, F 59 | 18 yrs |

**SUPPLEMENTARY TABLE 3S**

**COMPARISON OF DIAPASON DATA WITH OTHER STUDIES WHICH USED THE CAN**

|  | **USER-RATED UNMET NEEDS** | | | | |
| --- | --- | --- | --- | --- | --- |
| **CAN domains** | DIAPASON (%) | Cleary & Hunt, 2006 | Brunt & Hansson, 2002 | Grinshpoon & Ponizovsky, 2008 | Martinelli et al., 2022) |
| Basics |  |  |  |  |  |
| Accommodation | 9.0 (5.8; 12.2) | 7 (1.6; 12.4) | - | 7.7 (0.5; 14.9) | NA |
| Food | 7.0 (4.2; 9.8) | 8 (2.2; 13.8) | - | 5.8 (0; 12.2) | NA |
| Daytime activities | 8.3 (5.2; 11.4) | 28 (18.5; 37.5) | 13.7 (5.9; 21.5) | 19.2 (8.5; 29.9) | NA |
| Social |  |  |  |  |  |
| Company | 22.0 (17.4; 26.6) | 36 (25.8; 46.2) | 11.8 (4.5; 19.1) | 25 (13.2; 36.8) | NA |
| Intimate relationships | 22.0 (17.4; 26.6) | 54 (43.4; 64.6) | 15.7 (7.5; 23.9) | 25 (13.2; 36.8) | NA |
| Sex life | 17.6 (13.4; 21.8) | - | 5.9 (0.7; 11.2) | 26.9 (14.8; 39.0) | NA |
| Functioning |  |  |  |  |  |
| Basic education | 0.3 (0.0; 0.9) | 1 (0; 3.1) | 2 (0; 5.2) | 5.8 (0; 12.2) | NA |
| Money | 11.2 (7.7; 14.7) | 14 (6.6; 21.4) | 2 (0; 5.2) | 9.6 (1.6; 17.6) | NA |
| Childcare | 2.6 (0.8; 4.4) | 1 (0; 3.1) | 2 (0; 5.2) | 3.8 (0; 9.0) | NA |
| Self-care | 2.9 (1.0; 4.8) | 8 (2.2; 13.8) | - | 9.6 (1.6; 17.6) | NA |
| Living environment | 6.4 (3.7; 9.1) | 11 (4.3; 17.7) | 2 (0; 5.2) | 5.8 (0; 12.2) | NA |
| Health |  |  |  |  |  |
| Physical health | 5.8 (3.2; 8.4) | 9 (2.9; 15.1) | 13.7 (5.9; 21.5) | 13.5 (4.2; 22.8) | NA |
| Psychotic symptoms | 14.1 (10.2; 18.0) | 24 (14.9; 33.1) | 13.7 (5.9; 21.5) | 23.1 (11.6; 34.6) | NA |
| Drugs | 0 | 26 (16.7; 35.3) | - | 9.6 (1.6; 17.6) | NA |
| Alcohol | 0.3 (0; 0.9) | 13 (5.9; 20.1) | - | 3.8 (0; 9.0) | NA |
| Safety to self | 3.8 (1.7; 5.9) | 13 (5.9; 20.1) | 3.9 (0; 8.3) | 9.6 (1.6; 17.6) | NA |
| Safety to others | 0.6 (0; 1.5) | 13 (5.9; 20.1) | - | 15.4 (5.6; 25.2) | NA |
| Psychological distress | 22.7 (18.1; 27.3) | 28 (18.5; 37.5) | 9.8 (3.1; 16.5) | 25 (13.2; 36.8) | NA |
| Services |  |  |  |  |  |
| Information | 8.6 (5.5; 11.7) | 24 (14.9; 33.1) | 3.9 (0; 8.3) | 48.1 (34.5; 61.7) | NA |
| Telephone | 4.2 (2.0; 6.4) | 12 (5.1; 18.9) | - | - | NA |
| Transport | 9.6 (6.3; 12.9) | 2 (0; 5.0) | - | 11.5 (2.8; 20.2) | NA |
| Benefits | 8.6 (5.5; 11.7) | 2 (0; 5.0) | 3.9 (0; 8.3) | 36.5 (23.4; 49.6) | NA |
|  | **STAFF-RATED UNMET NEEDS** | | | | |
| **CAN domains** |  |  |  |  |  |
| Basics |  |  |  |  |  |
| Accommodation | 10.2 (6.8; 13.6) | 0 (0) | NA | NA | 9.3 (5.3; 14.9) |
| Food | 12.5 (8.8; 16.2) | 11 (4.3; 17.7) | NA | NA | 9.9 (5.8; 15.6) |
| Daytime activities | 18.9 (14.6; 23.2) | 46 (35.4; 56.6) | NA | NA | 20.5 (14.5; 27.6) |
| Social |  |  |  |  |  |
| Company | 39.0 (33.6; 44.4) | 49 (38.4; 59.6) | NA | NA | 38.5 (30.6; 46.9) |
| Intimate relationships | 31.6 (26.4; 36.8) | 40 (39.4; 60.6) | NA | NA | 51.3 (34.8; 67.6) |
| Sex life | 16.9 (12.7; 21.1) | - | NA | NA | 38.5 (20.2; 59.4) |
| Functioning |  |  |  |  |  |
| Basic education | 1.9 (0.4; 3.4) | 3 (0; 6.6) | NA | NA | 5.9 (2.7; 10.9) |
| Money | 18.9 (14.6; 23.2) | 46 (35.4; 56.6) | NA | NA | 21.3 (15.2; 28.4) |
| Childcare | 4.5 (2.2; 6.8) | 3 (0; 6.6) | NA | NA | 81.8 (48.2; 97.7) |
| Self-care | 10.2 (6.8; 13.6) | 6 (1.0; 11.0) | NA | NA | 23.0 (16.7; 30.3) |
| Living environment | 14.7 (10.8; 18.6) | 9 (2.9; 15.1) | NA | NA | 19.7 (13.6; 27.1) |
| Health |  |  |  |  |  |
| Physical health | 5.8 (3.2; 8.4) | 17 (9.0; 25.0) | NA | NA | 16.8 (11.4; 23.5) |
| Psychotic symptoms | 30.4 (25.3; 35.5) | 40 (39.4; 60.6) | NA | NA | 36.3 (28.8; 44.2) |
| Drugs | 1.3 (0; 2.6) | 31 (21.2; 40.8) | NA | NA | 1.9 (0.4; 5.3) |
| Alcohol | 1.0 (0; 2.1) | 20 (11.5; 28.5) | NA | NA | 1.9 (0.4; 5.3) |
| Safety to self | 1.6 (0.2; 3.0) | 40 (39.4; 60.6) | NA | NA | 9.9 (5.8; 15.6) |
| Safety to others | 1.6 (0.2; 3.0) | 17 (9.0; 25.0) | NA | NA | 3.8 (1.4; 8.0) |
| Psychological distress | 27.2 (22.3; 32.1) | 57 (46.5; 67.5) | NA | NA | 42.9 (35.1; 50.9) |
| Services |  |  |  |  |  |
| Information | 4.2 (2.0; 6.4) | 17 (9.0; 25.0) | NA | NA | 7.1 (3.6; 12.3) |
| Telephone | 6.4 (3.7; 9.1) | 0 (0) | NA | NA | 7.0 (3.5; 12.2) |
| Transport | 11.5 (8.0; 15.0) | 11 (4.3; 17.7) | NA | NA | 19.4 (13.5; 26.5) |
| Benefits | 4.2 (2.0; 6.4) | 0 (0) | NA | NA | 5.2 (2.3; 10.0) |

***References supplementary TABLE 2S***

1. Brunt, D., & Hansson, L. (2002). Comparison of user assessed needs for care between psychiatric inpatients and supported community residents*. Scand J Caring Sci, 16*(4), 406-413.
2. Cleary, M., & Hunt, G. E. (2006). The patient’s view of need and caregiving consequences: a cross-sectional study of inpatients with severe mental illness*. J Psychiatr Ment Health Nurs, 13(*5), 506-514.
3. Grinshpoon, A., & Ponizovsky, A. M. (2008). The relationships between need profiles, clinical symptoms, functioning and the well-being of inpatients with severe mental disorders. *J Eval Clin Pract, 14*(2), 218-225.
4. Martinelli, A., Iozzino, L., Pozzan, T., Cristofalo, D., Bonetto, C., Ruggeri, M. (2022). Performance and effectiveness of step progressive care pathways within mental health supported accomodation services in Italy. *Soc Psychiatry Psychiatr Epidemiol, 57*(5), 939-952.

**SUPPLEMENTARY TABLE 4**

**SOCIO-DEMOGRAPHIC AND CLINICAL PREDICTORS OF DIFFERENCES IN USER-RATED (CAN-P) AND STAFF-RATED (CAN-S) UNMET NEEDS. WE COMPUTED EACH DELTA AS CAN-P MINUS CAN-S**

|  | **Dependent variables** | | | | | |
| --- | --- | --- | --- | --- | --- | --- |
| **Predictors** | **∆Basic** | **∆Social** | **∆Functioning** | **∆Health** | **∆Services** | **∆Total** |
| Sex (Female) | 0.17  (-0.08; 0.41) | -0.08  (-0.32; 0.17) | 0.22  (-0.02; 0.46) | **0.27***  **(0.03; 0.51)** | 0.16  (-0.08; 0.40) | 0.21  (-0.03; 0.45) |
| Age (years) | -0.02  (-0.13; 0.09) | **-0.15****  **(-0.26; -0.04)** | -0.03  (-0.14; 0.08) | 0.03  (-0.08; 0.14) | -0.01  (-0.12; 0.10) | -0.07  (-0.18; 0.04) |
| Education level  (Elementary/junior high school) | -0.03  (-0.26; 0.19) | 0.03  (-0.20; 0.25) | 0.01  (-0.21; 0.24) | 0.06  (-0.17; 0.28) | 0.09  (-0.14; 0.31) | 0.05  (-0.18; 0.27) |
| Working status |  |  |  |  |  |  |
| Working (ref) | 0 | 0 | 0 | 0 | 0 | 0 |
| Studying | -0.21  (-0.82; 0.40) | 0.43  (-0.18; 1.04) | -0.01  (-0.62; 0.60) | 0.05  (-0.56; 0.66) | -0.16  (-0.77; 0.46) | 0.09  (-0.52; 0.70) |
| Not working | -0.16  (-0.50; 0.18) | 0.27  (-0.07; 0.61) | -0.20  (-0.53; 0.14) | -0.32  (-0.65; 0.02) | 0.05  (-0.29; 0.39) | -0.10  (-0.44; 0.24) |
| Support system^a^ | 0.08  (-0.03; 0.19) | 0.01  (-0.10; 0.12) | -0.03  (-0.14; 0.08) | -0.02  (-0.14; 0.09) | -0.02  (-0.13; 0.09) | 0  (-0.11; 0.11) |
| Collaboration skills^b^ | -0.04  (-0.15; 0.07) | -0.08  (-0.19; 0.04) | **-0.16****  **(-0.27; -0.05)** | -0.08  (-0.19; 0.03) | -0.01  (-0.12; 0.11) | **-0.12***  **(-0.23; -0.01)** |
| BPRS | -0.10  (-0.21; 0.01) | 0.02  (-0.09; 0.13) | **-0.17****  **(-0.28; -0.06)** | **-0.22*****  **(-0.33; -0.12)** | -0.03  (-0.14; 0.08) | **-0.16****  **(-0.27; -0.05)** |
| length of mental health disorder (years) | 0  (-0.11; 0.11) | **-0.16****  **(-0.27; -0.05)** | -0.04  (-0.15; 0.07) | 0.02  (-0.09; 0.13) | -0.03  (-0.14; 0.08) | -0.07  (-0.18; 0.04) |
| Length of stay in the RF (years) |  |  |  |  |  |  |
| <1 | -0.23  (-0.55; 0.08) | **0.54*****  **(0.23; 0.85)** | 0.11  (-0.21; 0.43) | 0.14  (-0.18; 0.45) | 0.13  (-0.18; 0.45) | 0.26  (-0.05; 0.58) |
| 1-5 | -0.19  (-0.44; 0.04) | **0.36 ****  **(0.12; 0.59)** | 0.04  (-0.20; 0.29) | 0.06  (-0.18; 0.31) | -0.22  (-0.46; 0.02) | 0.07  (-0.17; 0.32) |
| >5 (ref) | 0 | 0 | 0 | 0 | 0 | 0 |
| ^a^ Scores from 1 (family, friends and/or other people care about the patient; they are willing to cooperate and can do so) to 4 (absence of family, friends and other persons. Services should or should provide necessary support)  ^b^ Scores from 1 (actively seeks treatment, is well disposed and able to cooperate) to 5 (actively refuses treatments)  *p<.05  **p<.01  ***p<.001  Linear regression, standardized coefficients | | | | | | |
